# Inhibiting Shuttle Effect and Dendrite Growth in Sodium–Sulfur Batteries Enabled by Applying External Acoustic Field

**DOI:** 10.1021/acs.nanolett.4c00864

**Published:** 2024-08-21

**Authors:** Qipeng Zhang, Luyu Bo, Hao Li, Liang Shen, Jiali Li, Teng Li, Yunhao Xiao, Zhenhua Tian, Zheng Li

**Affiliations:** † Department of Mechanical Engineering, Virginia Polytechnic Institute and State University, Blacksburg, Virginia 24061, United States

**Keywords:** Sodium−sulfur battery, Acoustic field, Shuttle effect, BaTiO_3_, Piezoelectric effect

## Abstract

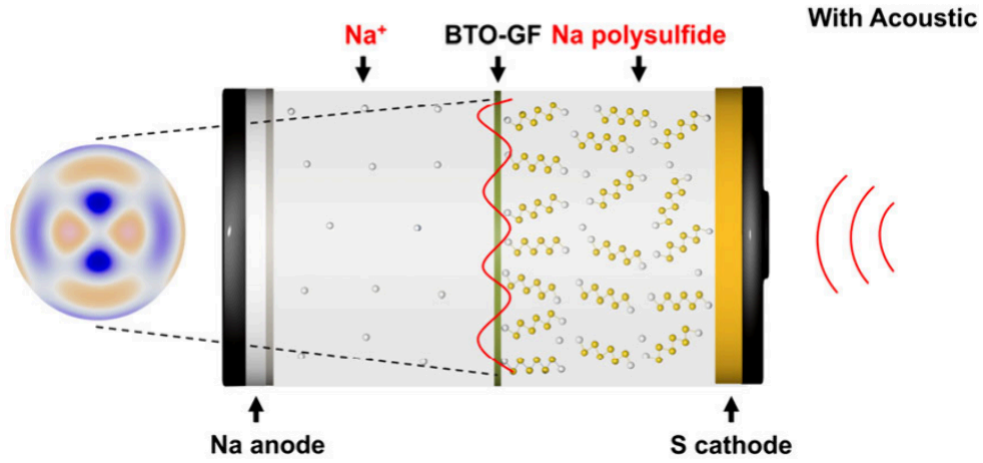

The room-temperature sodium–sulfur (RT Na–S) battery is a promising alternative to traditional lithium-ion batteries owing to its abundant material availability and high specific energy density. However, the sodium polysulfide shuttle effect and dendritic growth pose significant challenges to their practical applications. In this study, we apply diverse disciplinary backgrounds to introduce a novel method to stimulate polarized BaTiO_3_ (BTO) nanoparticles on the separator. This approach generates more charges due to the piezoelectric effect under stronger driving forces produced by applying a controllable acoustic field at the outer edge of the cell. The acoustically stimulated BTO attracts more polysulfides, thus reducing the shuttling effect from the cathode to the anode and ultimately enhancing the battery performance. Meanwhile, the acoustic waves create additional streaming flows, improving the uniformity of the sodium ion dispersion, enhancing the sodium ion transport and reducing the possibility of sodium dendrite development. We believe that this work offers a new strategy for the development of high-performance Na–S batteries.

The battery industry is exploring alternative energy-storage technologies beyond traditional lithium-ion batteries to reduce the cost, boost the energy density of electric vehicles (EVs), and facilitate renewable energy storage.^1−4^ Over the past decade, substantial research has been focused on the development of lithium–sulfur (Li–S) batteries. This interest is driven by the abundance of sulfur and the high theoretical capacity of 1675 mAh g^–1^, positioning Li–S batteries as promising candidates for application in EVs.^5−7^ However, sodium–sulfur (Na–S) batteries are the “dream technology” in terms of sustainability and economics because of their similar chemistry, comparative cost ($50–100 kWh^–1^), and the abundance of sodium compared to lithium.^8−12^

High-temperature sodium–sulfur batteries (HT Na–S), which utilize molten electrodes and a β-alumina solid electrolyte, have been developed for large-scale energy storage systems. However, these batteries’ working temperature (300–350 °C) significantly exceeds the melting points of sodium (98 °C) and sulfur (115 °C), which raises operation and maintenance expenses as well as safety issues.^13^ These drawbacks have driven interest in exploring room temperature sodium sulfur batteries (RT Na–S) for safer operation.^14^ A high theoretical specific energy of 1274 Wh kg^–1^ for RT Na–S has been reported since 2006.^15^ However, some technical issues, such as self-discharge, limited cycle life, and a low active material usage rate, hinder its growth. In addition, the poor compatibility between the sulfur cathode and electrolyte results in the high solubility of sodium polysulfide intermediates (NaPSs), particularly in ether-based electrolytes. As a result, sodium polysulfides shuttle to the sodium anode, leading to the loss of active substances, deterioration of interface, rapid capacity decay, and low Coulombic efficiency (CE).^13,14,16−18^ At the anode, on the other hand, the inhomogeneous growth and deposition of sodium dendrites pose significant safety issues, low CE, and poor cycle life.^12,19−21^ These challenges have greatly limited the practical applications of RT Na–S batteries.

To address these challenges, researchers have discovered compositing carbon-based hosts with sulfur and electrolyte modification.^22−25^ For instance, Pint et al. constructed a microporous confinement cathode using the processing of sucrose.^26^ The polysulfide shuttle on the cathode was reduced by the restraint method, which delivered more than 300 mAh g^–1^ at 1 C. Wang et al. formulated “cocktail optimized” electrolyte system that combined carbonate electrolytes, highly concentrated sodium salt, and indium triiodide as an additive.^14^ This tailored electrolyte in Na–S batteries exhibited outstanding performance with a specific capacity of 1170 mAh g^–1^ at 0.1 C.

In addition to the methods mentioned above, since the strong bonding affinity between anions/cations and polysulfides effectively inhibits the shuttle effects, it has been demonstrated that filling metal materials, such as oxides, sulfides, nitrides, and carbides, as well as their heterostructures composited with separator or cathode, can serve as polysulfide traps.^27−31^ Among these materials, the improved additive BaTiO_3_ (BTO) has gained increasing interest due to its potential to capture more polysulfides through ferroelectric effects.^32−35^ Wei et al. proposed a composite C/S cathode incorporating ferroelectric BTO materials, suggesting that BTO spontaneously polarizes under the action of an internal electric field. This polarization carried an electric charge on the surface that can absorb polar polysulfides. The cell had a discharge capacity of 835 mAh g^–1^ after 100 cycles, which is higher than its C/S equivalent without BTO.^36^ Chen et al. demonstrated that using a defective ferroelectric B-BTO as a multipurpose sulfur immobilizer improves the Li–S battery performance. The shuttle effect was both electrostatically and chemically constrained due to the inherent ferroelectricity of B-BTO and the chemical interactions between B-BTO and polysulfide molecules.^35^ The shuttle effect can be mitigated more effectively by leveraging the spontaneous polarization of BTO induced by a ferroelectric action. This approach is particularly effective in Li–S systems with additives such as LiNO_3_. However, it remains inadequate for Na–S systems, where the uncontrollable shuttle effect of sodium polysulfide is further exacerbated by the significantly higher solubility of higher-order NaPSs compared to the lithium polysulfide system during the multistep reaction process. This increased solubility of sodium polysulfide leads to a reduced cycling life.^37−39^ Additionally, sodium dendrites exhibit lower chemical stability compared with lithium dendrites, making it more difficult to find solutions to these combined issues.

Beyond the ferroelectric properties of BTO, the asymmetric crystal structure of BTO leads to a change in the distribution of positive and negative charges within the crystal when it is subjected to external mechanical stress, known as the piezoelectric effect. This change can be modified based on the intensity of the applied driving force. Consequently, the BTO’s piezoelectric action generates more polarization than the previously discussed ferroelectric effect (see Figure S1, Supporting Information). Based on this principle, it is feasible to apply an externally controllable field to the battery, inducing BTO to generate additional charges under stronger driving forces through the piezoelectric effect. This approach can attract more sodium polysulfides, thereby enhancing the electrochemical performance of the system using a common ether electrolyte without the additives.

In this paper, building on diverse disciplinary expertise, we propose the use of BTO nanoparticles coated onto commercially available glass fiber (GF) separators by a straightforward drop-coating procedure (Figure S2). To make the effect more obvious, we prepolarize the BTO. When exposed to an external acoustic field, the driving force stimulates the polarized BTO, resulting in an increased charge accumulation on the surface of the BTO nanoparticles due to the piezoelectric effect. This process enables BTO to absorb more sodium polysulfides and lessen the shuttling effect, enhancing the capacity of the Na–S battery to reach 300 mAh g^–1^ after 50 cycles compared to zero without acoustic waves. Additionally, to our surprise, the sodium metal anode is further stabilized by applying an external acoustic field compared with the base one by testing a symmetric Na–Na and long cycle Na–Cu cells. Finite element simulation indicates that the mechanical waves generated cause the electrolyte flow, improving sodium ion diffusion and lowering the likelihood of sodium dendrite formation in the sodium ion consumption region, thus stabilizing the battery performance.

Figure 1a depicts the X-ray diffraction (XRD) patterns of pure BTO, GF, and the polarized BTO-coated separator (referred to as BTO-GF). A broad band around 25° is observed, corresponding to the GF. The cubic (*Pm*3*m*) crystal system in which the XRD pattern for pure BTO is indexed in good accordance with those described in the literature.^40^ After the dropping and prepolarized process, no detectable impurities or new peaks are detected in the BTO-GF samples, indicating that the crystal system of BTO remains unchanged during this process. Additional SEM analysis of the BTO-GF after the dropping display a 2 μm thick GF with a homogeneous distribution of BTO nanocrystals (Figure S3).

**Figure 1:**
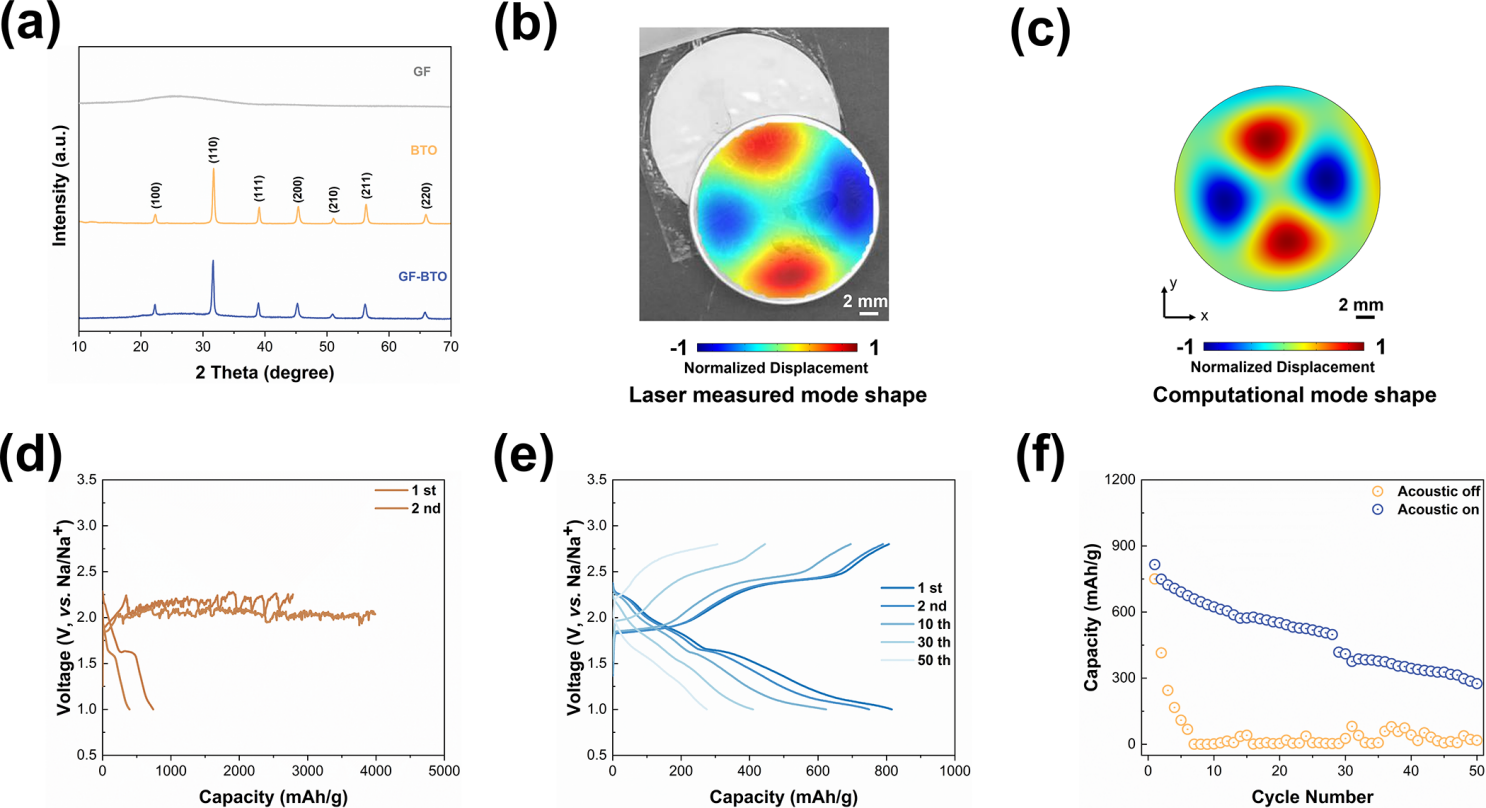
(a) XRD patterns of GF, BTO, and GF-BTO. (b) Laser measured mode shape of the battery. (c) Computational mode shape of the battery. (d) Voltage profiles of Na–S cells without acoustic field. (e) Voltage profiles of Na–S cells with acoustic field. (f) Cycling performance of the Na–S cells w/o an acoustic field.

To verify the impact of acoustic waves on mitigating the NaPSs shuttling issue and enhancing battery stability, Na–S cells were assembled and cycled both with and without an acoustic field. We created a small acoustic device to oscillate the battery. The device was made by a transducer and attached to the anode with a glass slide. The transducer could generate the 22 kHz sine wave, and the duty cycle was 50% (Figure S4). The vibration’s amplitude at this frequency is 0.5408 nm, which agrees with the computation (Figure 1b,c). The results of the Na–S battery are shown in Figure 1d–f, where two voltage plateaus are observed during the discharge process for both types of cells. The higher voltage plateau, around 2.2 V, corresponds to the solid–liquid transformation from S to the long-chain polysulfide Na_2_S_8_. The lower plateau, at approximately 1.6 V, represents the conversion from soluble Na_2_S_4_ to insoluble Na_2_S_*x*_ (*x* ≤ 3), a typical “solid-liquid-solid” conversion.^41^ For the charge curves, however, they are quite different. An undesirable, lengthy charging platform at about 2.2 V is noticed after the acoustic field is turned off. This is a sign of a significant NaPSs shuttling that leads to low reversibility and capacity deterioration.^42^ Therefore, the cell without an acoustic field displays poor discharge capacity dropping rapidly to almost zero after 10 cycles. In contrast, the cell with acoustic waves exhibits a complete charging curve, with two platforms at ∼1.8 and 2.2 V, corresponding to the conversion from lower-order to higher-order polysulfides, consistent with previously reported literature.^41^ The battery delivers a capacity of around 300 mAh g^–1^ after 50 cycles in the absence of additives, demonstrating a significant reduction in the shuttling effect and a clear improvement in both the longevity and reversible capacity. Additionally, XPS and EIS measurements were provided more evidence of decreased capacity from different perspectives, as shown in Figures S5 and S6, respectively. In the XPS testing, the Na–F peak at 683.2 eV, a crucial composition of the interphase layer, is observed under both battery conditions in the F 1s spectrum.^43^ However, a new peak corresponding to the TFSI anion signal at 687.8 eV emerged in the absence of an external acoustic field.^42^ This is likely due to the decomposition of sodium salts after numerous cycles, resulting in a decrease in capacity. Contrarily, the interphase layer composition under the application of an acoustic field reveal only one peak, showing that the external acoustic field could increase stability during cycling. Furthermore, the EIS measurements of cells with the acoustic field show lower interfacial resistance, with *R*_f_ and *R*_ct_ values of 254.6 and 768.1 Ω, respectively, compared to the cell without acoustic field, which exhibit *R*_f_ and *R*_ct_ values of 436.9 and 1637 Ω after 50 cycles.

SEM characterization and corresponding elemental mapping of BTO-GF and base batteries after 50 cycles were tested to provide further evidence supporting the previously discussed results. As shown in Figure 2, when the acoustic field is turned off, only a minor portion of sodium polysulfides are captured, indicating that the shuttling effect cannot be fully suppressed. However, as the electrochemical reaction proceeds, most sodium polysulfides are trapped on the glass fiber separator, suggesting that more sodium polysulfides are attracted to BTO in the presence of an acoustic field. Furthermore, the color changes observed in the sodium metal anode corroborate the SEM findings. We observe that when the acoustic field is turned off, a majority of sodium polysulfides are deposited on the surface of the sodium metal through the glass fiber separator, leaving only a small amount on the separator itself. This deposition turns the surface of the sodium metal yellow, as shown in Figure 2c. In contrast, the sodium metal in the battery exposed to the acoustic field retained a dull white color. This suggests that the acoustic field effectively inhibits the diffusion of sodium polysulfides to the anode side, as a significant amount of polysulfides are trapped within the BTO-GF separator.

**Figure 2:**
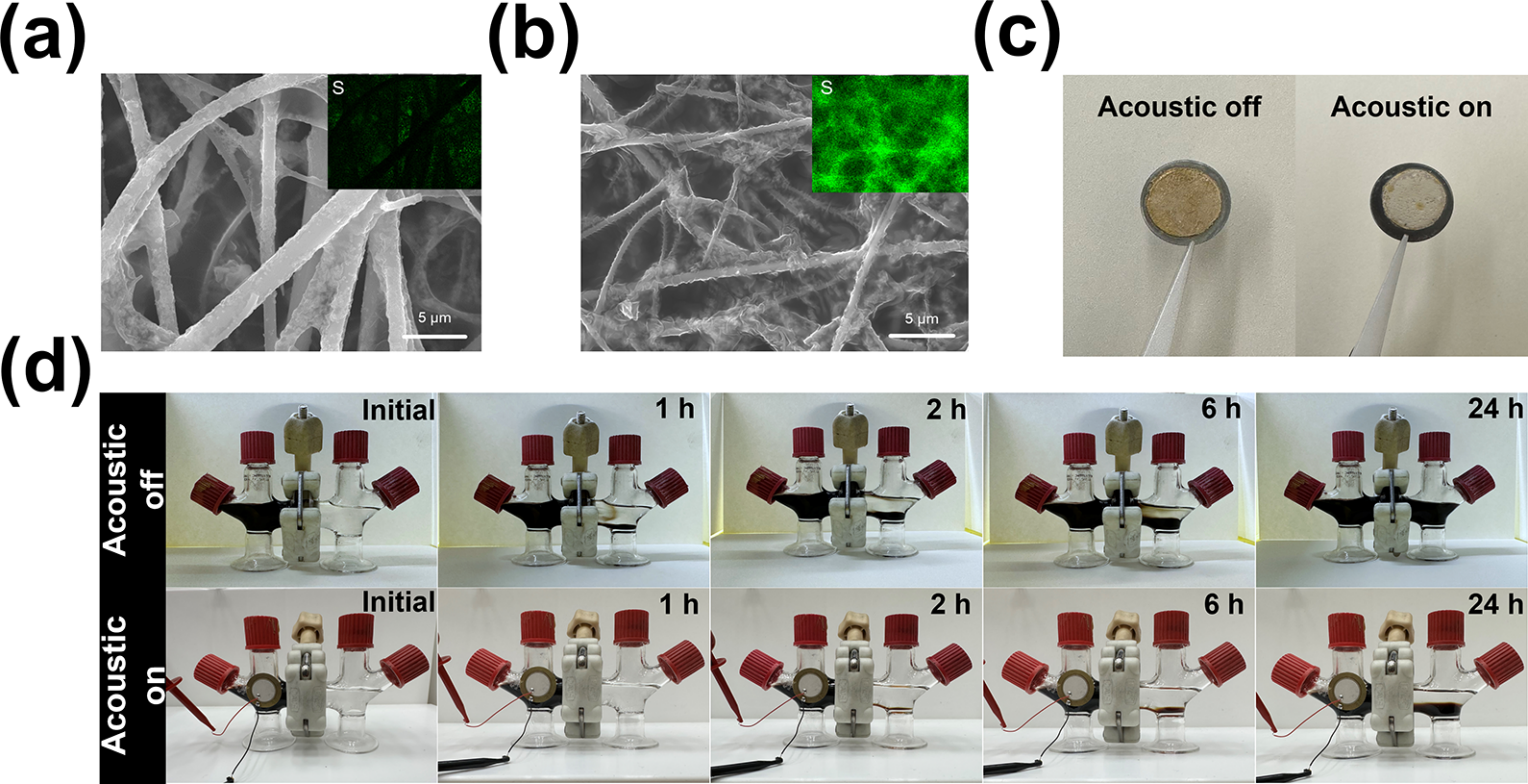
(a) SEM image of the cells without acoustic wave. (b) SEM image of the cells with acoustic wave. (c) Photo images comparing the sodium anodes without (left) and with (right) acoustic field. (d) Visual observation of NaPSs diffusion for the battery w/o acoustic field.

To directly identify the diffusion of soluble NaPSs in the presence or absence of an acoustic field, the permeability of NaPSs through a BTO-modified glass fiber separator was monitored by tracking color changes, as illustrated in Figure 2d. In the container without an acoustic field, significant migration of NaPSs to the opposite side is observed. After 1 h, sodium polysulfide diffuses across the container and settles at the bottom. By 24 h, the NaPSs have fully migrated to the other side. In contrast, under an acoustic field, no NaPSs migration is detected within the first hour. Only partial transfer of NaPSs is observed between 2 and 6 h. Approximately half of the NaPSs have migrated to the opposite side after 24 h. This shows that the container with an acoustic field effectively inhibits NaPSs diffusion. In addition, visual observation of NaPSs diffusion in the acoustic field using a GF without BTO was also conducted, as shown in Figure S7. Upon initial activation of the acoustic field, no migration of sodium polysulfide is observed. However, after 1 h, NaPSs begin to diffuse to the opposite side and settle at the bottom. By the 2 h mark, approximately half of the NaPSs have migrated to the other side, and by the 10th h, complete transition is achieved. This rate of transition is notably faster than in scenarios in which BTO is present, highlighting that the acoustic field alone cannot halt the diffusion of sodium polysulfides.

Finite element simulation was employed to comprehend why the majority of NaPSs are inhibited in the presence of an external acoustic field. The cathode and anode were located on either side of the separator and electrolyte (Figure 3a). An effort was made to match the vibrometer readings by modeling the boundaries at the acoustic transducer region as required displacement. With this model, we obtained the battery’s deformations at various eigenfrequencies by providing an oscillating excitation signal to the transducer. Our findings are consistent with the experiment (Figure 3b). Although the polarized BTO has a piezoelectric effect in the absence of an acoustic field, it is unable to produce a larger field force due to the formation of a weak composite field, as shown in Figure 3c. In contrast, when present in an external acoustic field, BTO nanoparticles are more polarized under stronger driving forces produced by coupled field, leading to the generation of more charges on the surface, which makes more NaPSs readily adsorbed, thus suppressing the shuttle effect and eventually enhancing performance, as shown in Figure 3d.

**Figure 3:**
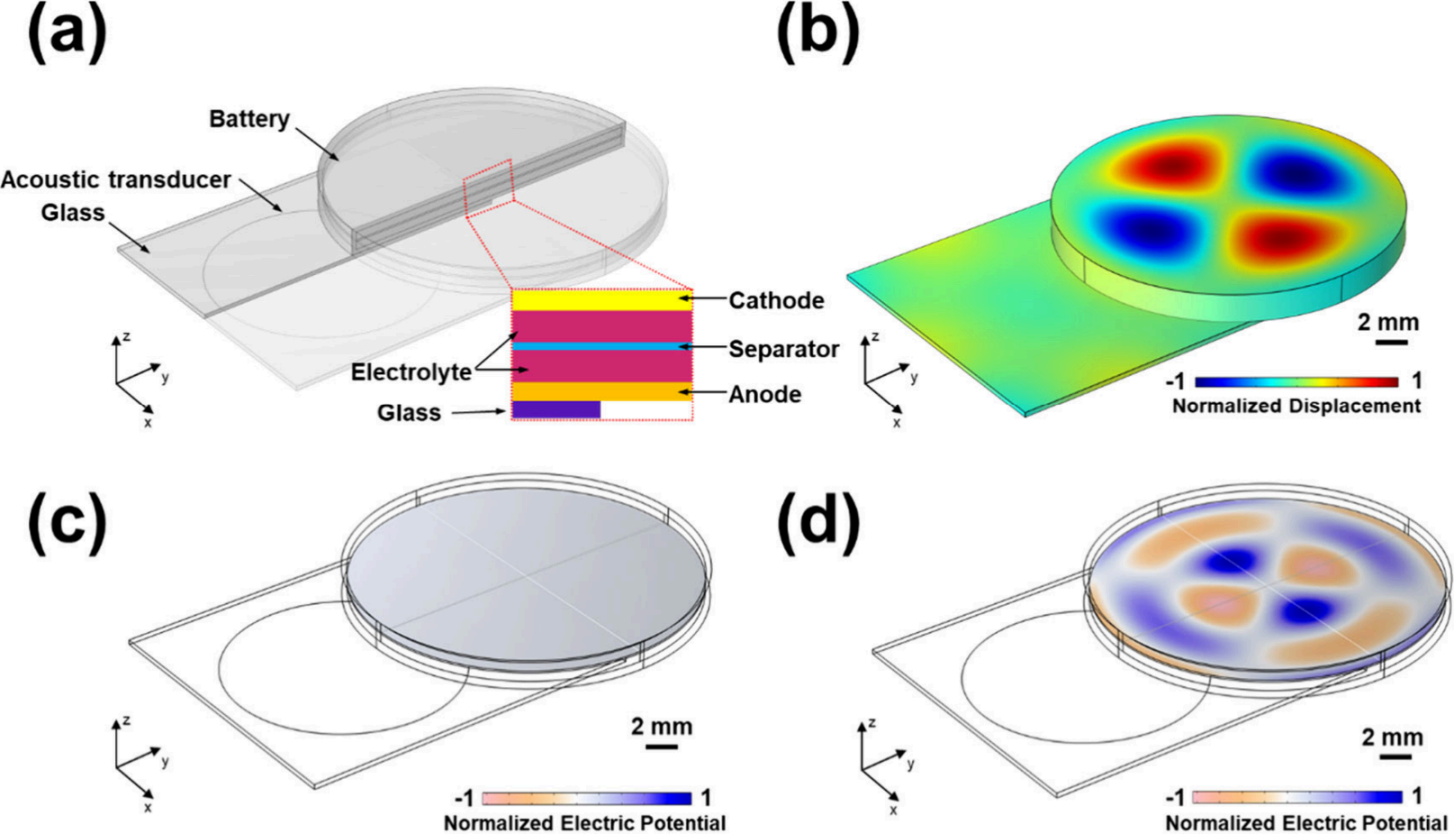
(a) 3D skematic of the battery model for finite element simulations. (b) Simulated mode shape of a cell. (c) Simulated electric fields for cases without an acoustic wave. (d) Simulated electric fields for cases with an acoustic wave.

While exploring the effect of the acoustic field on the sulfur cathode, a preliminary evaluation was also conducted to assess the impact of acoustic waves on the sodium metal anode. Although the research is not yet comprehensive, some initial observations are worth discussing. The average Na plating/stripping Coulombic efficiencies of Na–Cu and longer cycle life of Na–Na symmetric cells were evaluated on the impact of the acoustic field on the Na anode. As demonstrated in Figure 4a, drastic side reactions on the sodium anode surface result in a low average Coulombic efficiency (CE_avg_) of 61.57% in the absence of acoustic waves and additives. In contrast, when the acoustic field is applied, the CE_avg_ improved to 76.84%, suggesting that the acoustic field enhances the sodium plating/stripping behavior. Furthermore, the Na ion nucleation overpotentials were assessed (Figure S8). Without the acoustic field, the nucleation overpotential is 158.1 mV. In contrast, a much lower nucleation overpotential of 67.6 mV is observed with the acoustic field, further indicating improved sodium plating/stripping. The long-term cycling performance of Na–Na cells was tested to further evaluate the reversibility of sodium plating and stripping at a current density of 0.1 mA cm^–2^ and an areal capacity of 0.1 mAh cm^–2^ (Figure 4b). Cells without the acoustic field exhibit generally gradual increase in overpotentials with cycling time, reaching the cutoff voltage of 250 mV at 250 h, which is lower than those with cells using regular glass fiber separators (Figure S9). This difference may be attributed to the inherent piezoelectric properties of BTO, as the field generated by BTO can inhibit dendrites growth.^44^ In contrast, the voltage hysteresis of the cell with an acoustic field is further reduced and stabilized at around 110 mV without noticeable variations. We also employed finite element simulation to provide an explanation of how the external acoustic field influences the stability of sodium. In the absence of an acoustic field, inadequate diffusion leads to an uneven distribution of sodium ions within the anode, resulting in the irregular formation of sodium dendrites. These dendrites generate a strong electric field, which attracts additional sodium ions with each cycle, further accelerating their growth, as seen in Figure S10.^45^ However, the acoustic field can create an additional flow field, which provides an extra driving force (Figures 4c,d and S11), causing the electrolyte to flow. In order to simulate the acoustic streaming, the continuity and the Navier–Stokes equations are used, expressed as follows:^46,47^
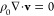
1
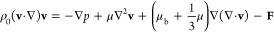
2where ρ_0_ is electrolyte density, **v** is streaming velocity, *p* is pressure, μ and μ_b_ are shear and bulk dynamic viscosities, respectively, and **F** is the body force, which can be expressed as^48^
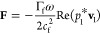
3where Γ_f_ is damping coefficient, ω is the angular frequency, *c*_f_ is the sound speed, *p*_1_ represents acoustic pressure, and **v**_1_ is the acoustic velocity. Numerical simulations were performed using the commercial finite element software COMSOL Multiphysics (see the Supporting Information for simulation procedures). The flow rate obtained based on our simulation is around 300 μm s^–1^, which is often higher than the electrolyte flow rate in the porous electrode.^49^ The presence of the flow rate enables more effective transport of sodium ions within the designated depletion zone, thereby slowing the formation of sodium dendrites.

**Figure 4:**
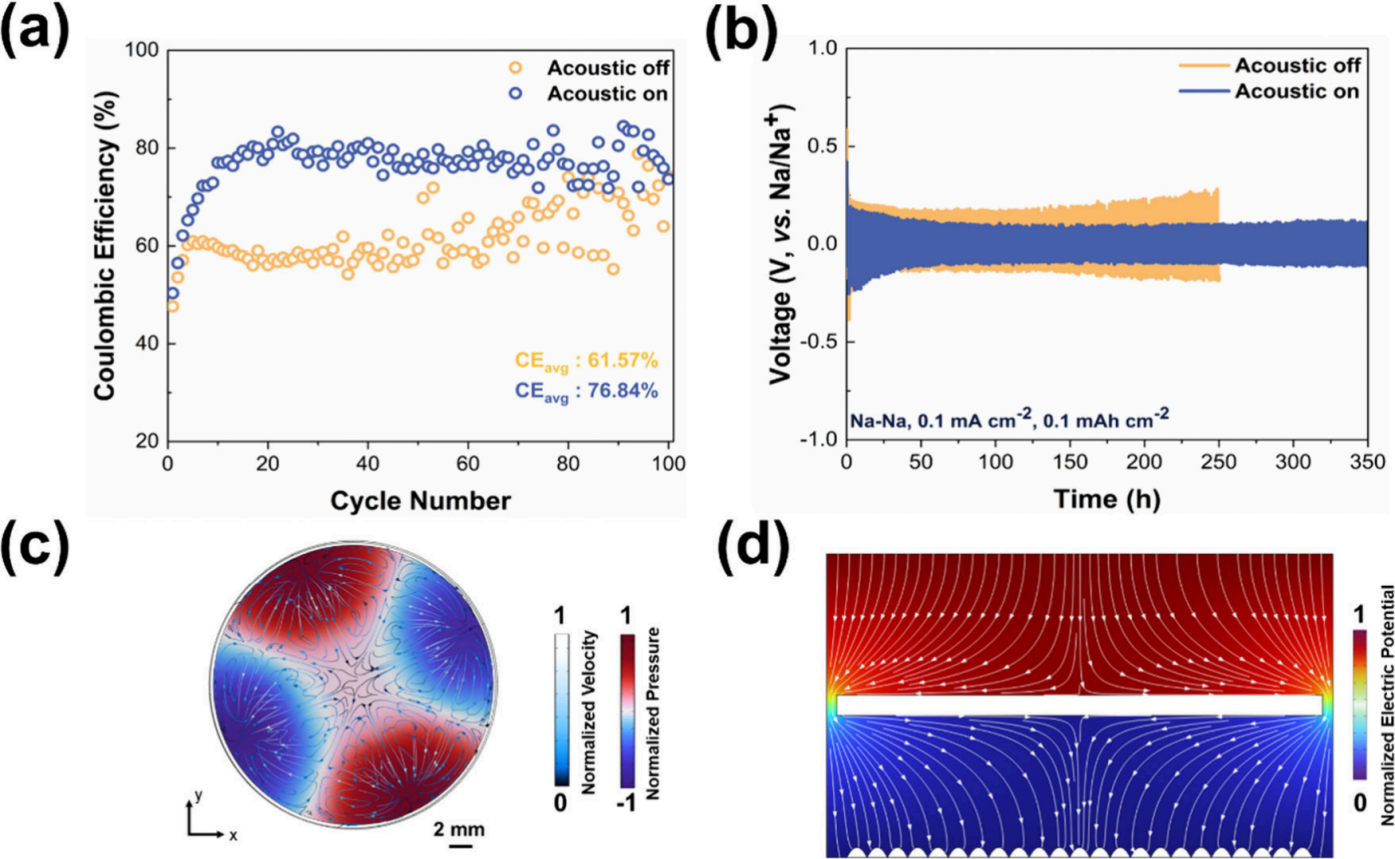
(a) Na–Cu cells using BTO-GF w/o acoustic field. (b) Voltage profiles of Na–Na symmetric cells w/o acoustic field. (c) Simulation results of velocity and pressure in the electrolyte with an acoustic wave. (d) Simulation results for electric field distribution of sodium dendrite with an acoustic wave.

In conclusion, an interdisciplinary method to control the shuttle effect of NaPSs by applying an external acoustic field to both sides of the battery case is proposed. Additional surface charges are generated when BTO nanoparticles are exposed to an external acoustic field, where these nanoparticles become more polarized under the stronger driving force created by coupled fields due to the piezoelectric effect. This increased surface charge attracts more sodium polysulfides, thus mitigating the shuttle effect. Sodium polysulfide transfer visualization experiments, along with comprehensive electrochemical characterizations and finite element simulations, provide strong evidence for the method’s validity and effectiveness. Furthermore, the enhanced streaming flows generated by the acoustic field promote a uniform distribution of sodium ions, reducing the risk of the likelihood of sodium dendrite formation. This dendrite growth inhibition is demonstrated through long-term stability tests, including Na–Na symmetric batteries and Na–Cu cells. The application of acoustic fields significantly improves the performance and cycle life of various battery systems. Given these beneficial effect advantages, we believe that this technology offers a novel approach to producing high-performance Na–S batteries.

## Supporting Information Available

Experimental section, figures showing details about polarization curves, SEM images, XPS spectra, EIS curves, voltage profiles of Na–Na symmetric cells, and simulation results (PDF)
