# Supplementary material for: Inhibiting Shuttle Effect and Dendrite Growth in Sodium–Sulfur Batteries Enabled by Applying External Acoustic Field
Source: Nano Lett. 2024 Aug 21;24(35):10711–7. doi: 10.1021/acs.nanolett.4c00864 (PMC11378336; doi:10.1021/acs.nanolett.4c00864)
Supplement: Supplementary file 1 — nl4c00864_si_001.pdf [file nl4c00864_si_001.pdf]

# Inhibiting Shuttle Effect and Dendrite Growth in Sodium-Sulfur Batteries Enabled by Applying External Acoustic Field

Qipeng Zhang,[†, §] Luyu Bo,[†, §] Hao Li,[†] Liang Shen,[†] Jiali Li,[†] Teng Li,[†] Yunhao Xiao,[†] Zhenhua Tian,[†, *] and Zheng Li[†, *]

[†]Department of Mechanical Engineering, Virginia Polytechnic Institute and State University, Blacksburg, Virginia 24061, United States, USA

*E-mail: tianz@vt.edu (Zhenhua Tian)    zhengli@vt.edu (Zheng Li)

§ Q.Z. and L.B. contributed equally to this paper

# 1. Experimental section

***Preparation of the BTO-contained separator.*** To prepare the BTO-contained separator, 1 g $BaTiO_3$ (BTO) nanoparticles (US Research Nanomaterials, Inc) was dispersed in 20 mL N-methyl-2-pyrrolidone (NMP) using a high-speed mixer at room temperature for 12 h. The BTO dispersion was then dropped in the glass fiber membrane separators (Whatman GF/A) and dried under vacuum overnight. The loading amount of the BTO particles was ~ 0.6 mg $cm^{-2}$.

***Preparation of the cathode.*** The sulfur/carbon (S/C) composite was prepared following a melt-diffusion strategy through mixing sulfur powder and mesoporous carbon powder (CMK-3, Xfnano, Inc.) in the weight ratio of 3:1, followed by heating the S/C mixture in a sealed vessel at 155 °C for 12 h under vacuum. The battery electrode slurry consists of S/C composite, Super P, and polyvinylidene fluoride (PVDF) binder with a mass ratio of 8:1:1 in NMP solvent. Then homogeneous slurry was cast onto the aluminum foil. All of the cathodes were dried at 60 °C in a vacuum for 12 h. The sulfur mass loading was about 1.0 mg $cm^{-2}$.

***Cell assembly and electrochemical testing.*** Electrochemical tests were performed using CR2032-type coin cell with sodium metal as the anode, BTO-contained membrane as the separator, the S/C composite as the cathodes, and 1.0 M bis(trifluoromethane)sulfonimide sodium salt (NaTFSI, Alfa Aesar) in tetraethylene glycol dimethyl ether / 1,3-dioxolane (TEGDME/DOL, 1:1 vol %) as the electrolyte. TEGDME and DOL were dried by 3 Å molecular sieves for over two weeks. Each cell contained 80 μL of electrolyte. The Na-S cells were galvanostatic cycled between 1.0 and 2.8 V on a LAND test system (Wuhan LAND Electronics Co., Ltd.) at room temperature (~ 26 °C). The cells were cycled at 0.1 C (1 C = 1675 mA $g^{-1}$). For Na-S cells with acoustic field, we apply two parallel one transducer to the cell case, which form stable acoustic field (**see Figure S4**)

Na-Na symmetric cells were tested at a current density of 0.1 mA $cm^{-2}$ with a fixed

areal capacity of 0.1 mAh cm$^{-2}$. The coulombic efficiencies of Na depositing/stripping were investigated via Na-Cu coin cells in corresponding electrolytes at a current density of 0.1 mA cm$^{-2}$ with a fixed areal capacity of 0.1 mAh cm$^{-2}$ using the method described by Zhang et al.[1] Plating a specific amount of Na metal onto a Cu substrate which is initially devoid of Na (represented by $Q_P$), Na metal is then stripped from the Cu substrate until a cut-off voltage of +1 V (shown by $Q_S$). The average CE over n cycles can be calculated as:

$$CE_{avg} = \frac{1}{n}\sum \frac{Q_s}{Q_p} (S1)$$

EIS measurements were performed Bio-Logic SP-150 potentiostat. All Na-S cells were measured using a 5-mV amplitude over the frequency ranging from 100 kHz to 10 mHz. ***NaPSs Permeability Tests.*** A volume of 5 mL of electrolyte was contained in an H-cell, and 5 mL of the NaPSs solution was contained in the other side. The NaPSs solution was prepared by adding S and sodium sulfide (Alfa Aesar) in electrolyte. A divider that was placed in between the two bottles served as their connection. The 24-hour monitoring of NaPSs migration.

***Material characterization.*** Structural characterization of BTO and BTO-contained separator were conducted by a Rigaku SmartLab XRD instrument using Cu Kα radiation. SEM were performed on a FEI Quanta 600 FEG environmental scanning electron microscope. To examine the elemental composition and distribution on the separator surface, EDX mapping images were also obtained. The composition of the interphase layer was examined using X-ray photoelectron spectroscopy (XPS, PHI Quantera SXM).

***Finite element simulation.*** Simulations were performed using the finite element method (FEM)-based software, COMSOL Multiphysics. Our model was established according to the 3D schematic of the device (**Figure.3a**) containing a battery, a silica glass, and an acoustic transducer. The piezoelectric module was applied to the separator (*i.e.*, containing BTO), and the fluid domain (*i.e.*, electrolyte) was simulated with the

pressure acoustics module. The circular region (radius, 11.5mm) at the bottom of the silica glass was set to the “boundary load” condition, and the remaining boundaries were set to the “free load” condition. Then, the model was solved through COMSOL’s frequency domain analysis to obtain the displacement on the battery’s surface, the electrical potential on the surface of the separator, and the pressure in the fluid domain.

In order to simulate the streaming pattern in the fluid domain, the laminar flow physics module was used. The body force stemming from acoustic pressure, as described in Eq. (2), was calculated based on the simulated acoustic pressure. The no-slipping condition was set to all boundaries surrounding the fluid domain. Then, the model was solved by a “stationary” solver to obtain the streaming velocity in the electrolyte. [2, 3]

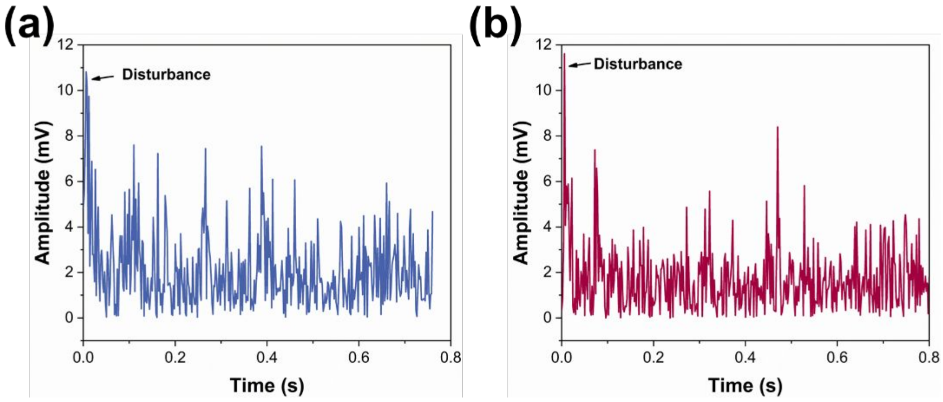


**Figure S1**. Comparative analysis of BTO nanoparticle polarization. (a) Cubic. (b) Tetragonal.

For two different crystal systems, we provide a disturbance for the BTO. For cubic, the polarization produced by the piezoelectricity is the consequent amplitude when a disturbance occurs because the polarized BTO is only piezoelectric. There is roughly a 10.8 mV amplitude. Since the polarized BTO in tetragonal crystal system exhibits both ferroelectricity and piezoelectricity, the amplitude is the superposition of these two phenomena. The amplitude is about 11.6 mV. This indicates that the polarization produced by piezoelectricity for a polarized BTO is significantly larger than the polarization produced by ferroelectricity.

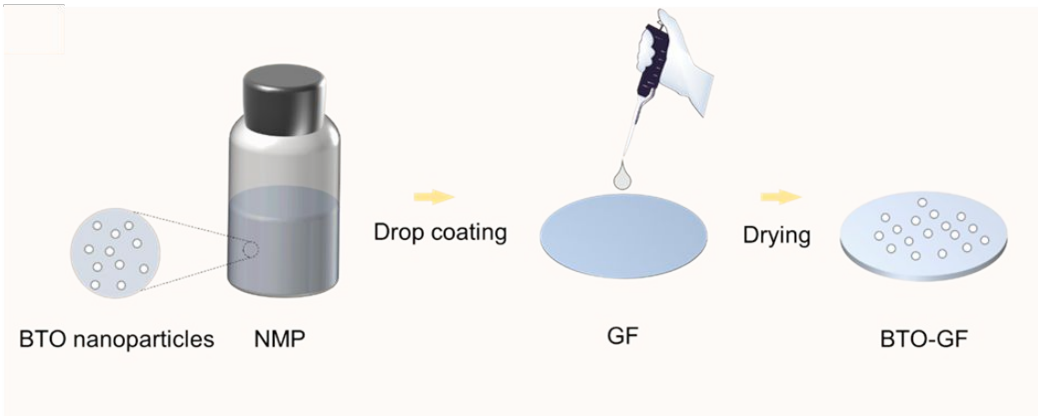


**Figure S2**. Schematic illustration on the preparation procedure of the BTO-coated separator by a drop coating process.

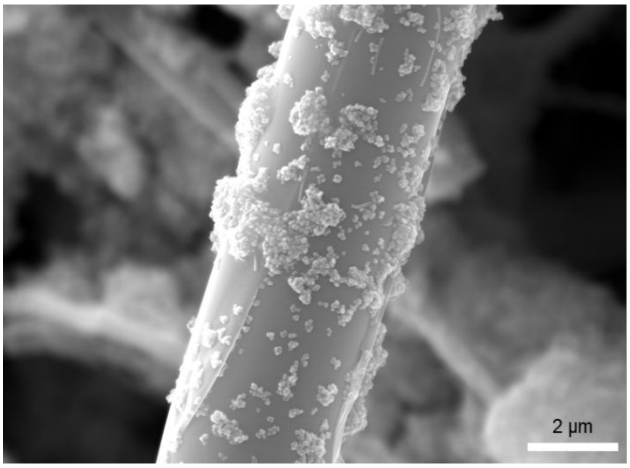


**Figure S3**. SEM images of the BTO-GF.

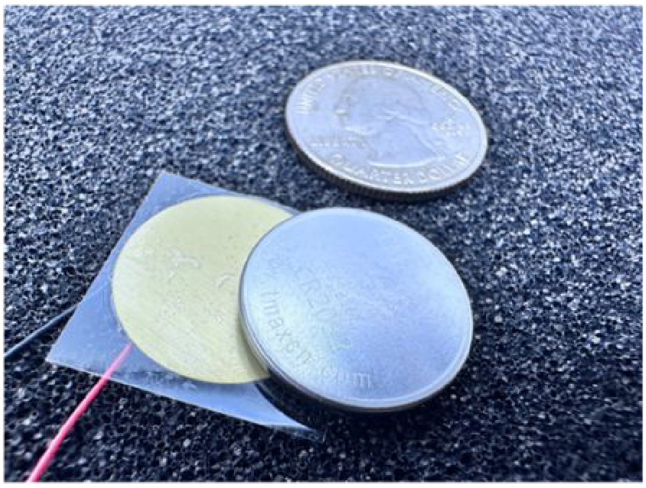

**Figure S4**. Photo of the test setup with a piezoelectric transducer and a battery bonded on a glass wafer.

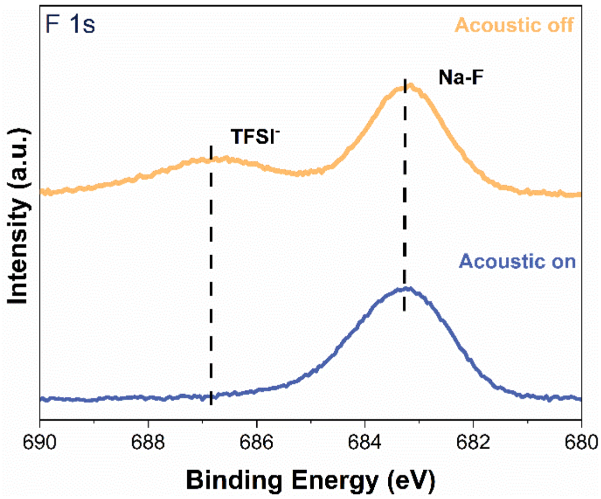


**Figure S5.** XPS spectra of cycled cell.

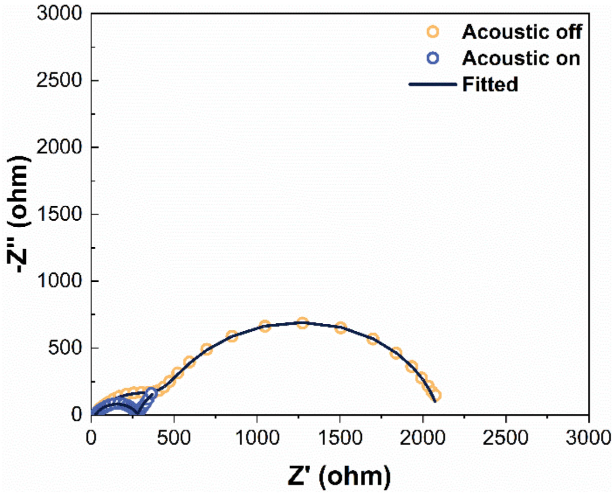


**Figure S6.** Electrochemical impedance spectroscopy (EIS) of Na-S cells with/without acoustic field after 50 cycles.

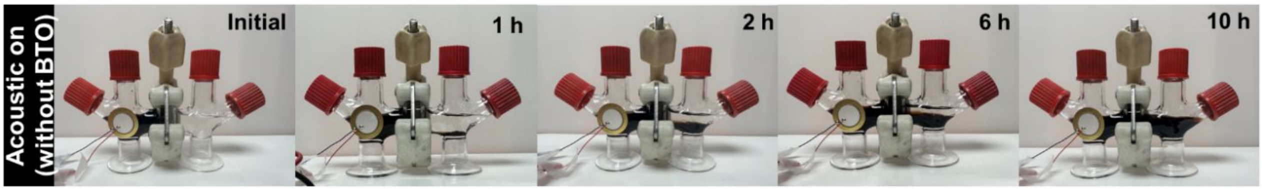

**Figure S7** Visual observation of NaPSs diffusion for battery in the acoustic field using GF without BTO.

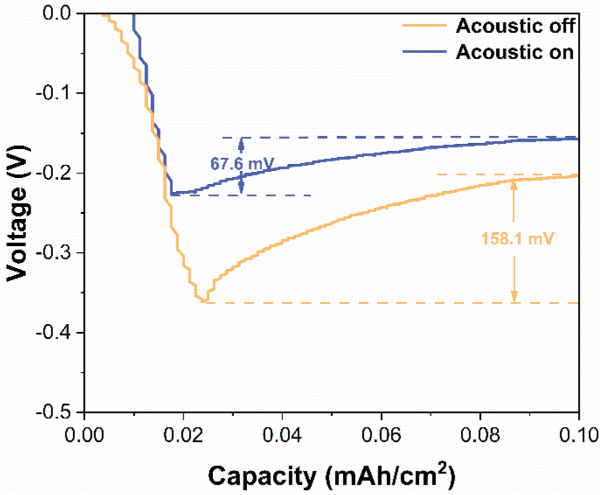


**Figure S8.** Comparison of the nucleation overpotentials for Na-Cu cells batteries w/o acoustic field.

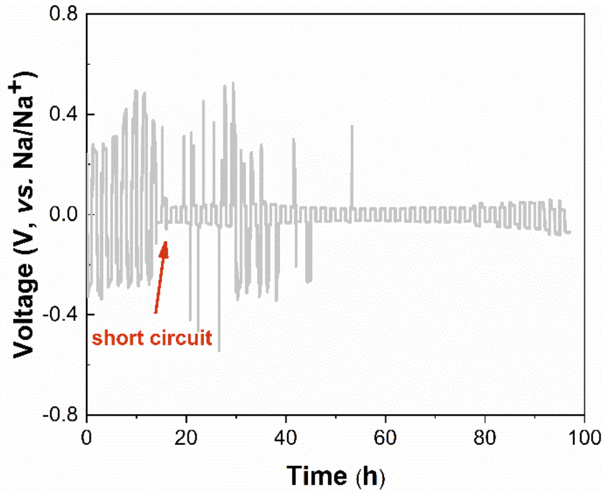


**Figure S9. V**oltage profiles of Na-Na symmetric cells using regular GF.

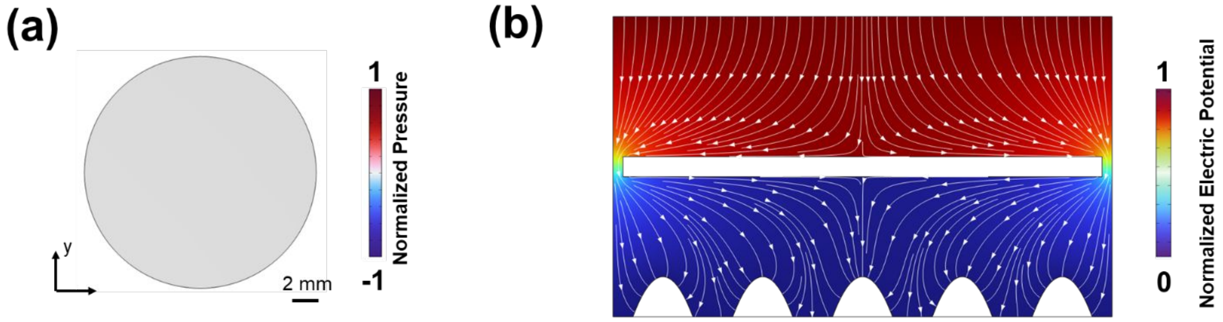


**Figure S10.** (a) Simulation result for the battery when acoustic waves are off. (b) Simulated electric field distribution in a region near the sodium dendrite when acoustic waves are off.

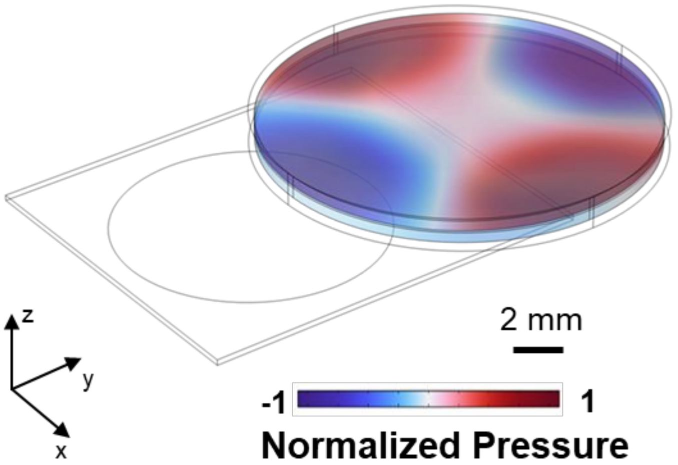


**Figure S11.** Simulated acoustic pressure in the electrolyte.

## Reference

1. Adams, B. D.; Zheng, J.; Ren, X.; Xu, W.; Zhang, J. G., Accurate Determination of Coulombic Efficiency for Lithium Metal Anodes and Lithium Metal Batteries. *Advanced Energy Materials* **2017,** *8* (7). DOI: 10.1002/aenm.201702097.
2. Shen, C.; Lu, S.; Tian, Z.; Yang, S.; Cardenas, J. A.; Li, J.; Peng, X.; Huang, T. J.; Franklin, A. D.; Cummer, S. A., Electrically Tunable Surface Acoustic Wave Propagation at MHz Frequencies Based on Carbon Nanotube Thin-Film Transistors. *Advanced Functional Materials* **2021,** *31* (18). DOI: 10.1002/adfm.202010744.
3. Giurgiutiu, V., *Structural Health Monitoring with Piezoelectric Wafer Active Sensors*. Elsevier: 2007.
